# Acetanilide in Enteric Fever

**Published:** 1890-03-22

**Authors:** 


### THERAPEUTICS.

### ACETANILIDE IN ENTERIC FEVER.

This drug has lately been advocated in the treatment of typhoid. The action seems to be almost entirely confined to reducing temperature. The usual dose is five to ten grains, given when the temperature reaches 101 deg. F., and repeated eight times a-day, if necessary. The use of acetanilide may be compared with that of quinine, salicylate of soda, antipyrin and antifebrin, all of which are given to reduce temperature in the same disease. None of them cut short the malady, but under their controlling action both the comfort and the safety of the patient are vastly increased.

